# LncRNAs and their role in cancer stem cells

**DOI:** 10.18632/oncotarget.22161

**Published:** 2017-10-30

**Authors:** Shusen Chen, Jiamin Zhu, Feng Wang, Zhifeng Guan, Yangyang Ge, Xi Yang, Jing Cai

**Affiliations:** ^1^ Department of Radiation Oncology, Nantong Tumor Hospital, Affiliated Tumor Hospital of Nantong University, Nantong, 226321, China; ^2^ Department of Radiation Oncology, Fudan University Shanghai Cancer Center, Department of Oncology, Shanghai Medical College, Fudan University, Shanghai, 200032, China

**Keywords:** cancer stem cells, lncRNA, cancer biology

## Abstract

Cancer stem cells (CSCs) play a vital role in the formation of tumors and have been studied as a target of anticancer therapy. Long non-coding RNAs (lncRNAs) are important in the genesis and progression of cancer. Various lncRNAs, such as ROR, HOTAIR, H19, UCA1, and ARSR, are involved in cancer stemness. These lncRNAs could regulate the expression of CSC-related transcriptional factors, such as SOX2, OCT4, and NANOG, in colorectal, prostate, bladder, breast, liver, and other cancer types. In this work, we review the progress of lncRNAs and cancer stem cells and discuss the potential signal pathways of lncRNAs in cancer stemness.

## INTRODUCTION

About 150 years ago, Virchow and Cohnheim first proposed the theory that cancers originate from a small population of stem cells and believed that “dormant embryonic tissue remnants” could be reactivated as cancer stem cells (CSCs); these cells consist of only a fraction of neoplastic cells, which were initially recognized as the hematopoietic system [1]. Since then, CSCs have been found in different solid tumors, such as liver cancer, breast cancer, glioma, and lung cancer [2–5]. CSCs are postulated as the “seeds” of tumor because of their metastasis and recurrence capacity [6]. CSCs possess numerous functions, such as initiating, maintaining, and developing cancer growth. Moreover, CSCs possess self-renewal ability, thus, tumors can grow morphologically and functionally diverse cells containing therapy-resistant and metastatic cell populations [7]. Highly specific biomarkers are needed to identify CSCs and investigate their molecular patterns. Information on CSCs in a tumor can be obtained using the following substitute markers: Notch family members, CD133 (prominin-1), CD24, CD44, Bmi-1, nestin, aldehydedehydrogenase1 (ALDH1), and Hedgehog [8]. These signaling factors play crucial roles in maintaining CSC characteristics. Numerous studies prove that CSCs are important contributors toward drug resistance, cancer metastasis, and recurrence following chemotherapy in tumors [9, 10].

LncRNAs constitute a class of non-coding RNAs with lengths exceeding 200 nucleotides, in contrast to miRNAs and siRNAs, lncRNAs cannot be translated into a protein [11]. At present, accumulated data reveal that lncRNAs are significantly involved in the control of multiple cellular processes. The epigenetic, transcriptional, or post-transcriptional gene regulation mechanisms could modulate the development, homeostasis, pluripotency, growth, and apoptosis of cells and metastasis of cancer [12–15]. LncRNAs are expressed aberrantly in various types of human cancers, such as colorectal, prostate, breast, liver, brain cancer, renal, and bladder cancer [16], in addition, numerous lncRNAs are thought to be associated with almost all types of cancers. For instance, MALAT-1, HOTAIR, H19, GAS5, UCA1, and GHET1 [17] could regulate the critical pathways of oncogenic and tumor suppression [18]. The dysregulation of lncRNAs is a potential biomarker in diagnosis, prognosis, and target therapy of cancers [19]. Although these lncRNAs are promising markers for cancers, their application involves an invasive procedure with possible complications, high cost, and susceptibility to sampling errors [20]. LncRNAs can affect the cancer development, such as tumorigenesis, apoptosis, metastasis, chemoresistance, radioresistance and angiogenesis, through various pathways, such as p53, KLF4-KRT6/13, Wnt6, and PI3 kinase/CREB pathways. In particular, maternally expressed gene 3(MEG3) is an important angiogenic lncRNA, which was investigated to be silenced in pituitary adenomas. MEG3 stimulated the pathways of p53 and regulated the target genes of p53 [21]. Recently, numerous studies revealed that various lncRNAs could regulate CSCs in numerous types of cancer via different molecular mechanisms, which involve differentiation, proliferation and self-renewal, promotion of the metastasis, invasion and prediction of prognosis and targeted therapies. To date, ROR, HOTAIR, H19, UCA1 and ARSR are the most highlighted lncRNAs in CSCs. In the following sections, we will discuss the relationship between the lncRNAs and CSCs in detail.

## ROR

LncRNA ROR is 2.6 kb in length. Pluripotent stem cells could be induced by reprograming differentiated cells. Importantly, ROR straightly targets major transcription factors, such as SOX2, OCT4, and NANOG, which are necessary for pluripotent stem cell phenotypes through the colocalization of the three factors in close proximity to the OR promoter region [13]. On the other hand, DNA and stem cell self-renewal could be damaged by ROR [22, 23].

Numerous studies have determined that ROR could act as a marker of cancers. ROR is observed to be markedly upregulated in various cancers, such as hepatocellular, breast, endometrial, pancreatic cancers and nasopharyngeal carcinoma [24, 25].

Chen et al. proved that ROR can indicate the multidrug resistance of breast cancer by eliciting epithelial–mesenchymal transition (EMT) [26], which is relevant to 5-FU and paclitaxel resistances in human malignant tumors [27–29]. Besides, in pancreatic cancer cells, ROR could mediate migration and metastasis partly by activating ZEB1 through inhibiting p53 expression [25]. ZEB1 can suppress the members of miR-200 family, leading to activation of EMT and maintenance of pancreatic cancer stemness [30].

ROR possibly performs unidentified functions by the regulation of gastric cancer stem cell (GCSC) differentiation and self-renewal. Several studies indicated that the enhancement of ROR expression in GCSCs regulated the expression of CSC-related transcriptional factors. Moreover, the CD133-mediated invasion and proliferation of GCSCs may be attributed to ROR [31].

MTT and EdU assays were conducted to determine whether ROR controls the growth of GCSCs. Interesting, the overexpression of ROR in CD133-cells importantly increased proliferation of cells relative to the vector control. On one hand, when ROR was knocked down, the cell growth rate of CD133+ cells could be markedly attenuated. Similarly, EdU analysis showed that, compared with the vector control, CD133-cells with ROR overexpression included an upper percentage of S-phase cells, whereas the reduction of ROR in CD133+ cells showed a considerably lower potential in inducing cell proliferation compared with the scramble control. All of these outcomes strongly support that ROR induces the proliferation of GCSCs [31].

## HOTAIR

HOTAIR is one of lncRNAs with a length of 2.2 kb. HOTAIR is transcribed from the antisense strand of HOXC gene cluster present in chromosome 12 [32].

Given that its overexpression is observed in numerous human malignant tumors, HOTAIR offers a hopeful approach for anti-cancer treatment. First, HOTAIR can be used as a tumor biomarker for cancer diagnosis. High expression levels of HOTAIR are related to enhanced metastasis, indicating a negative prognostic factor for the survival rate of patients. In various types of tumors, monitoring of the levels of HOTAIR can be used to predict the alterations in gene or protein levels, the risk of tumor development and progression, and the prognosis of the tumor. By studying murine xenograft models, researchers found that HOTAIR knockout can reduce the growth of tumor *in vivo* [33, 34]. Therefore, the knockout of the HOTAIR gene or decreasing the protein level of HOTAIR could provide a hopeful target for cancer therapy.

EMT is a cellular process that is generally associated with CSCs. Alves et al. evaluated the function of HOTAIR in EMT and the maintenance of CSCs. Importantly, HOTAIR regulates genes involved in EMT. Various studies also proved that HOTAIR regulates various genes associated with the progression of cell cycle, cellular structural integrity, cell–cell signaling and development. PCDHB5, ABL2, JAM2, PCDH10, SNAIL (snail family zinc), PRG1 (P53-Responsive Gene 1) and laminin HOXD10 are target genes of HOTAIR [33–38].

Several studies showed that HOTAIR knockdown can repress TGF-β1, which induces EMT and reduces the colony-forming ability of colon cancer cells. Compared with the non-stem cell subpopulations, the colon cancer stem cell subpopulation (CD133 (+)/CD44 (+)) possesses higher levels of HOTAIR, suggesting that HOTAIR aids in carcinogenesis via the acquisition of stemness. HOTAIR was also demonstrated to suppress the tumor inhibitor miR-7 by regulating the expression of HoxD10, thereby sustaining the expression levels of c-myc, TWIST and miR-9 and maintaining the EMT process and the CSC pool of breast cancer [39].

Recently, lncRNA was found to be commonly upregulated in hepatocellular carcinoma (HCC). In addition, HOTAIR was found to promote the growth of human liver CSC by limiting the association of P300, CREB and RNA pol II to the SETD2 promoter region, thereby restricting SETD2 phosphorylation and expression [31]. Therefore, HOTAIR not only exhibits oncogenic activity but also indicates poor tumor prognosis [40].

HOTAIR plays an important role in eliciting the transformation of CSCs in lung cancer. The generation of CSCs from human bronchial epithelial cells could be induced by cigarette smoke extract. HOTAIR is revealed to participate in the transformation process, for example, the depletion of HOTAIR could weaken spheroid formations and the percentage of side population enrichment. HOTAIR may induce the expression of BMI1, CD44, OCT4, and CD133. The expression of these genes is critical in the gene network reprogram to obtain CSC properties [41, 42].

As a crucial nucleotide molecule, single lncRNA usually performs multiple roles in various organisms. As a well-known lncRNA, HOTAIR is found to function as a modular scaffold for histone modification proteins, thus targeting special genes and promoting the metastasis of cancer [15].

In addition, substantial proof indicates that HOTAIR may participate in CSC regulation. In the colon CSC subpopulation, HOTAIR was discovered to be expressed at a markedly higher level (CD133+CD44+) compared with other non-stem cancer cells. Moreover, the knockdown of HOTAIR by siRNA is correlated with a decreased colony forming capacity of colon and breast cancer cells. These studies indicate that HOTAIR may be a critical regulator of cancer cell plasticity and a valuable predictor of tumor progression. HOTAIR inhibition may be a potential option for cancer prevention and CSC targeted therapies [43].

## H19

LncRNA H19 is an important and a maternally expressed gene. Humans and mice both possess the H19 gene. H19 performs an important role in regulating cell differentiation and proliferation [44]. In addition, H19 is an estrogen-regulated transcript. The aberrant expression of H19 leads to the proliferation and migration of various cancers, such as gallbladder, gastric, and pancreatic cancers [45, 46].

LncRNA feedback loops control SOX2, OCT4, and c-myc [13, 47]; thus, lncRNA could maintain the phenotypes of CSCs. Consistent with this idea, the suppression of H19 with siRNA in prostate epithelial cells (RWPE-1) reduces the colony-forming potential. By contrast, enhancement of H19 expression markedly increases sphere-forming capacity [48]. H19 promotes the formation of soft-agar colony in breast cancer cells. Studies uniformly revealed that breast CSCs (BCSCs)-enriched populations and breast tumors show high H19 expression. *In vitro*, function analysis further validated that H19 is crucial for BCSC properties. Moreover, a previous study revealed that the dysregulation of H19 could facilitate breast tumor progression after the subcutaneous injection of H19-recombined cells into SCID mice [49]. In xenograft nude mice model, studies demonstrated that H19 promotes tumor growth and tumor-initiating ability. However, evidence revealed that neither depletion nor overexpression of H19 influences cell proliferation in breast tumors, indicating that spheroid formation and anchorage-independent colony formation, as well as tumor-initiating abilities, controlled by H19 are not linked to proliferation and self-renewal [50].

## UCA1

Urothelialcarcinoma associated 1 (UCA1) is a 2314-bp lncRNA, which is encoded on human chromosome 19. UCA1 was identified in bladder cancer for the first time [51].

Numerous studies showed that UCA1 expression was upregulated in numerous types of cancers, including hepatocellular cancer, gastric cancer, colorectal cancer, lung cancer and esophageal squamous cell carcinoma. High UCA1 expression was strongly correlated with clinicopathologic characteristics, such as overall survival and lymph node metastasis [52–57]. One of the potentialities of CSC originates from the transformation of benign stem cells. UCA1 is demonstrated to possibly facilitate the malignant transformation of liver stem cell through the modification of the gene network by tail modification of histone. A research described that UCA1 restrains the trimethylation of H3K27. H3K27 is a marker of suppressive histone at the promoter of several genes, such as albumin, SOX17, HGF, HNF4α and FOXa2 in hepatocyte-like stem cells.

UCA1 also induced the expression of HULC and β-catenin, which contribute to the malignant growth hepatocyte-like stem cells [58]. The overexpression of UCA1 results in the increased binding of UCA1 to cyclin D1. The complex activates lncRNA H19 transcription via induction of DNA demethylation. High H19 expression could increase the cell telomerase activity by increasing the binding of TERT to TERC and severing the interaction between TERT and TERRA, which enhances the length of telomere. UCA1 also controls the expression of the pluripotency regulator c-myc. The UCA1–cyclin D1 complex is recruited to the c-myc promoter by insulator CTCF, resulting in the increase in c-myc expression [59]. Besides, UCA1 associates with SET1A to cause the malignant transformation of hepatocyte-like stem cell by enhancing cell proliferation. UCA1 enhances the phosphorylation of pRB1, which could promote the interaction between SET1A and pRB1. Then, the complex would induce the trimethylation of H3K4 on a specific gene promoter, including TRF2, an important factor in telomere extension [60]. Telomere length extension and c-myc expression are believed to be critical in the malignant transformation of hepatocyte-like stem cells. This result indicates that UCA1 may be involved in malignant transformation and could be a vital therapeutic target in the future [42].

## ARSR

The lncRNA Activated in RCC with sunitinib resistance (ARSR) was recently identified as a novel lncRNA. ARSR lies in chromosome 9q21, which is 591 nucleotides in length. ARSR increases the sunitinib resistance of renal cell carcinoma (RCC) [61].

Qu et al. investigated that ARSR is highly expressed in primary renal CSCs and predicts poor prognosis. Loss-of-function analysis in CSCs and gain-of-function analysis in RCC cells prove that ARSR possesses self-renewal capacity and oncogenicity and can promote the metastasis of renal CSCs. Further research on mechanisms reveals that ARSR interacts with Yes-associated protein (YAP) to block its phosphorylation by LATS1, thus promoting YAP nuclear translocation. Interestingly, YAP is found to consequently facilitate the transcription of ARSR, forming a feed-forward loop. In general, ARSR promotes the expansion of renal CSCs through interaction with YAP [62].

Accumulating evidence demonstrated that the CSCs of clear cell renal cell carcinoma (ccRCC) surviving from drug therapy and giving rise to cancer regrowth may be a major cause of therapeutic resistance [63–66]. The expression signature of stem cells or targets of OCT4, NANOG, c-myc(NOSM), and SOX2 in human ESCs(embryonic stem cells)were evidently enriched in the mRNA profile of sunitinib-resistant RCC cells(GSE69535), facilitating investigations of the function of ARSR in renal CSCs [62]. Existing information indicates that upregulation of the expression of ARSR in pre-therapy RCC tumors are importantly correlated with a poor sunitinib response. However, patients with low ARSR expression demonstrated attractive improvement in prognosis after receiving sunitinib. Hence, ARSR expression in RCC cancers is suitable for assessment to determine patients who may profit from sunitinib treatment prior to deciding on a treatment course. In terms of treatment-naïve patients with high expression of ARSR or activated AXL/c-MET, the combination with anti-lncARSR or anti-AXL/c-MET therapeutics may promote the response rate to sunitinib treatment. Thus, these data provides biological fundamentals for ARSR as a clinical indicator and therapeutic target for sunitinib resistance of RCC [61, 67].

## OTHERS

LncRNA–Hh contributes to stem cell renewal property. In breast cancer, this gene is regulated by TWIST. Specifically, the shh-GLI1 pathway is promoted by Hh to induce OCT4 and SOX2 expression in breast cancer cells, which is crucial in TWIST-induced maintenance of mammosphere enrichment and self-renewal capacity, this finding is similar to the tumorigenicity of breast cancer cells *in vivo* [68].

LncTCF7 is a critical participant in mediating HCC CSC maintenance and renewal. TCF can enhance the expression of NANOG, SOX2, and OCT4. LncTCF7 function in the role of lncRNA in cancer progression and development causes TCF7 expression by soliciting SWI/SNF complex to TCF7 promoter for transcription. Both TCF7 and TCF7 expression were observed in the sphere formation of liver cancer cells, indicating their importance in CSCs. Subsequently, the Wnt signaling of cancer cells could be activated by TCF7. Several studies showed that lncTCF7-induced Wnt signaling induces the tumorigenicity of HCC by controlling the self-renewal of liver CSCs [69].

Besides, LncRNA-34a promotes the self-renewal of (colon cancer stem cells) CCSCs. Moreover, Lnc34a asymmetrically induces cell fate asymmetry in the division of CCSC. This influence is mediated by miR-34a, which targets Wnt signaling and Notch pathways, which are necessary for CCSC self-renewal [70, 71].

Another lncRNA, MALAT1, regulates complex biological processes, such as synaptogenesis [72], the invasion of trophoblasts into the uterine wall [73] and tumor metastasis [74], by interacting with the serine/arginine-rich splicing factors. *In vitro* and *in vivo* experiments, MALAT1 indicates a pro-tumor function in the proliferation, invasion, and migration of cholangiocarcinoma cells. Accumulating evidence demonstrated that lower OS rate, poorer TNM stage, larger tumor size and metastasis in patients with hilarcholangiocarcinoma (HCCA) are correlated with the high expression levels of MALAT1. Hence, lncRNA MALAT1 is a highly promising novel prognostic tissue biomarker in HCCA.

In addition, lncRNA cancer-upregulated drug resistant (CUDR) can be found in numerous tumors owing to overexpression. CUDR can also promote tumorigenesis [58]. CUDR also exhibits considerable potential in controlling liver CSC via the cascade of CUDR–HULC/CUDR–B-catenin signaling pathways [31].

Interestingly, lncRNA p21 has been indicated as an effective allayer of the stem-like characteristics of CSCs isolated from colorectal cancer cell lines or primary colorectal cancer tissues [75]. Several reports stated that lncRNA-p21 attenuates the self-renewal and viability of colorectal cancer by inhibiting the B-catenin signaling [31].

## CONCLUSION

In this study, lncRNAs and their role in cancer stem cells are discussed. LncRNAs cannot be translated into a protein [11] but are critical in pathway of oncogenic and tumor suppression [18]. lncRNAs can influence the cancer development, such as tumorigenesis, apoptosis, metastasis, chemoresistance, radioresistance and angiogenesis. On the basis of previous studies, we can reasonably assume that lncRNAs are critical regulatory elements in the epigenetic modification of CSCs. We have reviewed that lncRNAs are defined to be associated with TNM stage, response rate to therapy and prognosis, considerable evidence indicates that lncRNAs will be critical markers for cancer therapeutics and diagnostics [31].

Further studies on lncRNA are needed to increase the understanding of the complex networks involved in controlling stem cells. These investigations may discover the mechanisms by which differentiated cancer cells can destroy developmental or embryonic programs to induce cancer cell self-renewal. In CSCs, future studies on lncRNAs, including profiling of classified cell populations of cancer and genetic approaches for navigating lncRNA expression, should provide information on the mechanism of these molecules controlling CSCs. Ongoing *in vivo* work will provide the strongest proof for the importance of lncRNAs in disease and physiology. In addition, further tumor profiling should recognize which lncRNAs may function as clinical biomarkers and are the best candidates for future therapeutic strategies. Functional studies on lncRNAs may be useful for the value of serum lncRNAs as indicators of different kinds of cancers. In total, further studies are necessary to improve the understanding of the complex networks involved in lncRNA and CSCs.
